# Methylation decreases the Bin1 tumor suppressor in ESCC and restoration by decitabine inhibits the epithelial mesenchymal transition

**DOI:** 10.18632/oncotarget.14914

**Published:** 2017-01-31

**Authors:** Xuexiao Wang, Jiali Wang, Yunlong Jia, Yu Wang, Xiaonan Han, Yuqing Duan, Wei Lv, Ming Ma, Lihua Liu

**Affiliations:** ^1^ Department of Biotherapy, Fourth Hospital of Hebei Medical University and Hebei Cancer Institute, Shijiazhuang, China; ^2^ Research Center, Fourth Hospital of Hebei Medical University and Hebei Cancer Institute, Shijiazhuang, China

**Keywords:** bridging integrator-1 (Bin1), methylation, esophageal squamous cell carcinoma (ESCC), Decitabine, epithelial mesenchymal transition (EMT)

## Abstract

Bridging integrator-1 (Bin1), as a tumor suppressor, is frequently attenuated or even abolished in multiple primary cancers. A reduced expression of Bin1 caused by DNA methylation, has been reported in breast and prostate cancers. However, the methylation status of Bin1 and potent biological functions in esophageal squamous cell carcinoma (ESCC) remain unclear. In a previous study, we showed that the Bin1 expression was low in ESCC tissues. Herein, we further characterized this mechanism, confirming that gene hypermethylation was significantly correlated with the aberrant attenuation of Bin1. In addition, the Bin1 hypermethylation was associated with the poorer clinical parameters and shorter survival times of ESCC patients. Methylation-specific reverse transcription-polymerase chain reaction (MS-RT-PCR) showed that Bin1 was hypermethylated in several ESCC cell lines, which might be the main cause of reduced Bin1 expression. In addition, treatment with the de-methylation agent Decitabine (DAC) could restore Bin1 expression and evidently restrained ESCC cell malignant behaviors, particularly the epithelial mesenchymal transition (EMT) via reactivating the PTEN/AKT signaling pathway to inhibit matrix metalloproteinase (MMP)-2 and MMP-9 expression *in vitro* and *in vivo*. In conclusion, these results demonstrated that Bin1 methylation could augment the malignant biological behaviors of ESCC and predict the poor prognosis for ESCC patients, thus indicating the potential clinical application value of DAC-based de-methylation therapy in ESCC.

## INTRODUCTION

Globally, esophageal cancer (EC) accounts for 5% of all malignant tumor deaths and is the eighth most common cancer and the sixth leading cause of cancer-related deaths worldwide [1]. In China, the majority of the EC is squamous cell carcinoma (ESCC), accounting for more than 90% of such cases [2]. Despite of the widespread use of multimodality therapies, the overall survival of the patients is still unsatisfactory. However, the underlying mechanisms are largely unknown [3]. Studies have increasingly indicated that the reduced expression of some tumor suppressor genes could be one of the major causes of ESCC tumorigenesis [4]. Therefore obtaining more knowledge of the associated mechanisms of aberrant tumor suppressor gene expression in ESCC tumorigenesis is urgently needed to initiate new therapeutic strategies.

Growing evidence indicates that aberrant epigenetic alterations play an important role in carcinogenesis and cancer progression [4]. DNA methylation is an enzymatic process involving the addition of a methyl group to the 5′-position of the pyrimidine ring of cytosines to produce 5-methylcytosine. This covalent moderation is catalyzed by DNA methyltransferases (DNMTs) in short CpG-rich DNA stretches known as CpG islands. CpG islands overlap the promoter area and the promoters may become aberrantly hypermethylated, causing transcriptional inhibition, in terms of reduction of gene expression [5–7]. Therefore, identifying the methylation status of tumor suppressor genes is necessary to realize the mechanisms involved in ESCC formation and progression.

Bridging integrator-1 (Bin1), also known as Amphiphysin 2 or SH3P9, has been involved in multiple diseases and affects different tissues and physiological functions [8]. Bin1 exerts tumor suppressing effect through several mechanisms, such as suppressing tumor cell migration, arresting cell cycle and stimulating apoptosis; however, Bin1 is commonly attenuated or even lost in multiple malignant carcinomas, such as lung cancer, prostate cancer, breast cancer and melanoma [9–11]. As the number of studies on Bin1 increases, its significance in carcinogenesis is gradually being recognized. In a previous study, we found that Bin1 was attenuated in ESCC tissues and the low expression of Bin1 was associated with poor clinicopathological parameters and predicted poor prognosis for ESCC patients [12]. Ekaterine et al. revealed that Bin1 deficiency might be a consequence of epigenetic alterations, such as methylation, which had been reported in prostate and breast cancers [13]. However, the methylation status of Bin1 in ESCC remains unclear; hence the effect of Bin1 methylation on ESCC carcinogenesis and tumor progression is also unknown. In the present study, we identified the methylation status of Bin1 in ESCC tissues and cell lines, and evaluated the relationship between the reduced expression of Bin1 and the methylation status of Bin1 gene. Then, we identified the prognostic roles of Bin1 methylation for ESCC patients. Furthermore, we evaluated the biological functions of Bin1 methylation in ESCC by employing *in vitro* and *in vivo* experiments, aiming at to lay a foundation for further studies on Bin1 in ESCC.

## RESULTS

### Association between Bin1 expression and methylation status revealed that the methylation of Bin1 was related to poor prognosis of ESCC patients

**Figure 1:**
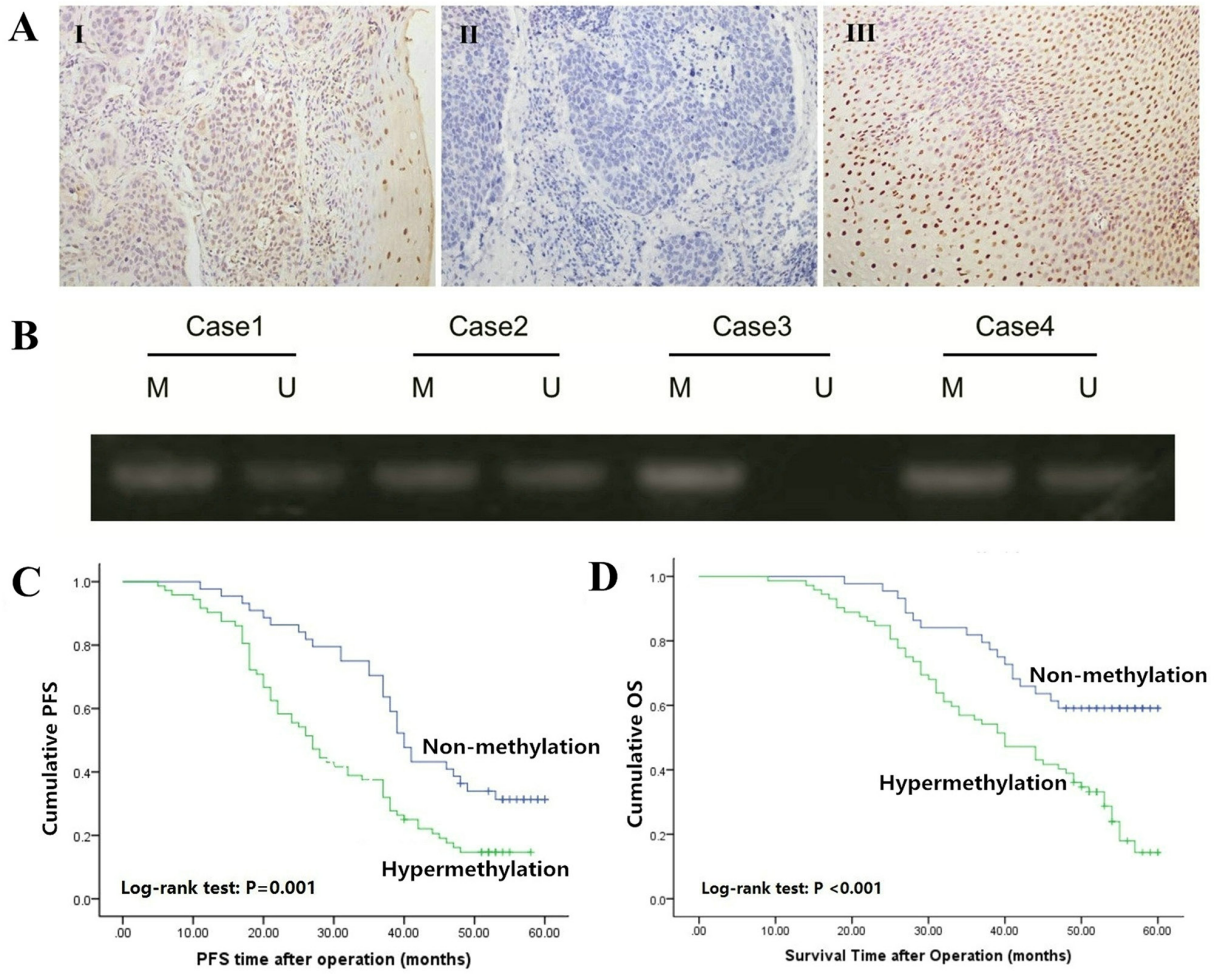
Expression and methylation status of Bin1 in ESCC tissues **A**. Immunohistochemical staining of Bin1 in ESCC tumor tissues and corresponding normal tissues (SP ×200). I positive staining of Bin1 in ESCC tissue; II negative staining of Bin1 in ESCC tissue; and III positive staining of Bin1 in para-carcinoma tissue. **B**. The methylation status of the promoter region of Bin1 determined through MSP analysis in ESCC tumor tissues. M for methylated, U for unmethylated. **C**. Correlation between Bin1 methylation in tumor tissue and PFS time of ESCC patients. **D**. Correlation between Bin1 methylation in tumor tissue and poor OS time of ESCC patients.

To reveal the protein expression pattern of Bin1 in ESCC patients, we detected the expression in carcinoma and para-carcinoma tissues with IHC. The staining of Bin1, which occurred in the cell nucleus, was observed in normal esophageal epithelium cells and squamous cancer cells (Figure 1A). The results demonstrated that the low-expression rate of Bin1 was significantly higher in carcinoma tissues than in para-carcinoma tissues [70/116 (60.34%) *vs*. 28/116 (24.14%), *P* < 0.001]. We further analyzed Bin1 expression at gene level using qRT-PCR and found that 64 cases of ESCC tissues exhibited a low expression of *Bin1* mRNA, accounting for 91.43% low Bin1 protein expression, indicating that the gene expression and protein expression were of consistent (Table 1).

**Table 1:** Comparison between the methylation status of Bin1 and clinicopathological features of ESCC patients

Clinicopathological Feature	n	Methylation frequency		
		n (%)	χ^2^	*P*
Gender				
Male	81	49(60.49)	0.283	0.595
Female	35	23(65.71)		
Age				
≤60	62	35(56.45)	1.785	0.182
>60	54	37(68.52)		
Differentiation				
Well+Moderate	52	26(50.00)	5.831	0.016
Poor	64	46(71.86)		
TNM stage				
I+II	90	49(54.44)	9.915	0.002
III+IV	26	23(88.46)		
Invasion depth				
T1+T2	44	20(45.45)	8.311	0.004
T3	72	52(72.22)		
Lymph node metastasis				
Positive	76	53(69.74)	5.504	0.019
Negative	40	19(47.50)		

We further detected the methylation status of *Bin1* mRNA using MSP to confirm whether the hypermethylation of Bin1 occurred in ESCC. The results demonstrated that among the 70 tumor tissues with low *Bin1* mRNA expression, the hypermethylation of Bin1 could be observed in 59 cases (92.19%), accounting for 84.29% low Bin1 protein expression (Figure 1B).

To further distinguish the effect of the Bin1 methylation status of the promoter region on ESCC carcinogenesis, we analyzed the relationships between Bin1 methylation status and ESCC clinicopathological parameters. The expression of methylated Bin1 was significantly related with the TNM stage, tumor differentiation grade, invasion range, and lymph node metastasis status but not with gender and age (Table 1). ESCC patients with poor differentiation grade, high TNM stage (stage III+IV), deep tumor invasion (T3), and positive lymph node metastasis had significantly higher rate of methylated Bin1 than did those with well or moderate differentiation grade, low TNM stage (stage I and II), superficial tumor invasion (T1 and T2), and negative lymph node metastasis (Table 1).

To confirm the significance of the Bin1 methylation status of the promoter region in predicting the prognosis of ESCC patients, we used Kaplan-Meier analysis and log-rank test to investigate Bin1 associations with progression free survival (PFS) and overall survival (OS). Univariate analysis indicated that the factors significantly associated with PFS were Bin1 methylation status, TNM stage, invasion depth, tumor differentiation grade and lymph node metastasis (all *P* < 0.01), whereas age and gender were not related to the PFS time of ESCC patients (*P* = 0.762; *P* = 0.499). Kaplan-Meier analysis showed that the PFS time of patients with Bin1 methylation was significantly shorter than that without methylation (*P* = 0.001; Figure 1C). In addition, univariate analysis indicated that the factors significantly associated with OS were Bin1 methylation status, TNM stage, invasion depth, tumor differentiation grade and lymph node metastasis (all *P* < 0.001), whereas age and gender were not related to the OS time of ESCC patients (*P* = 0.971; *P* = 0.994). Kaplan-Meier analysis indicated that the OS time of patients with methylation of Bin1 was significantly shorter than did those without methylation (*P* < 0.001; Figure 1D).

To further assess the predictive roles of methylated Bin1 in ESCC prognosis, the methylation of Bin1 promoter region and main clinicopathological parameters were included in the multivariate analysis using the Forward LR method. Covariates included in the Cox proportional hazards model were gender, age, differentiation grade, invasion range, lymph node metastasis, TNM stage, and Bin1 methylation status. After stepwise multivariate survival analysis, the differentiation grade, TNM stage, lymph node metastasis, Bin1 methylation were observed to be independent prognostic factors for ESCC patients (Table 2).

**Table 2:** Multivariate analysis of the correlation between clinicopathological parameters and survival time of ESCC patients

Variables	Multivariate analysis				
	Coefficient	Standard Error	HR	95% CI for HR	*P*
Methylation status (positive *vs*. negative)	0.750	0.290	2.116	1.199-3.735	0.016
Lymph node metastasis (positive *vs*. negative)	0.932	0.401	2.540	1.158-5.572	< 0.001
TNM stage (I+II *vs*. III+IV)	0.458	0.222	1.582	1.024-2.443	< 0.001
Differentiation (high *vs*. low+medium)	0.676	0.257	5.550	3.096-9.948	0.009

Bin1 loss was correlated with gene hypermethylation and DAC-induced Bin1 de-methylation could inhibit proliferation, arrest cell cycle and promote apoptosis of ESCC cells

**Figure 2:**
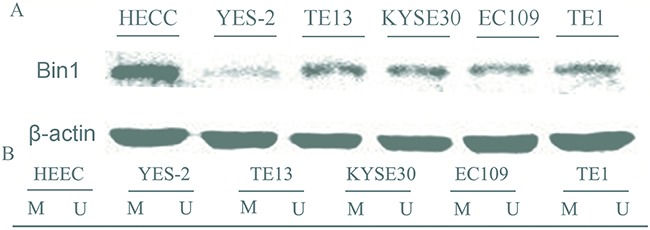
Expression and methylation status of Bin1 in five human esophageal cancer cell lines and human normal esophageal epithelial cells (HEECs) **A**. Protein expression of Bin1 in esophageal cancer cell lines and HEECs detected by western blot. **B**. The methylation status of Bin1 detected by MSP analysis in various cancer cell lines and HEECs. M for methylated, U for unmethylated.

To verify the expression status of Bin1 in ESCC cells, we evaluated Bin1 expression in five ESCC cell lines (YES-2, TE13, KYSE30, EC109 and TE1) and HEEC. Western-blot analysis indicated that Bin1 protein expression was high in HEECs, but was low in these five ESCC cells (*P* < 0.01) (Figure 2A). The expressions in these 6 cell lines was consistent at gene and protein level.

**Figure 3:**
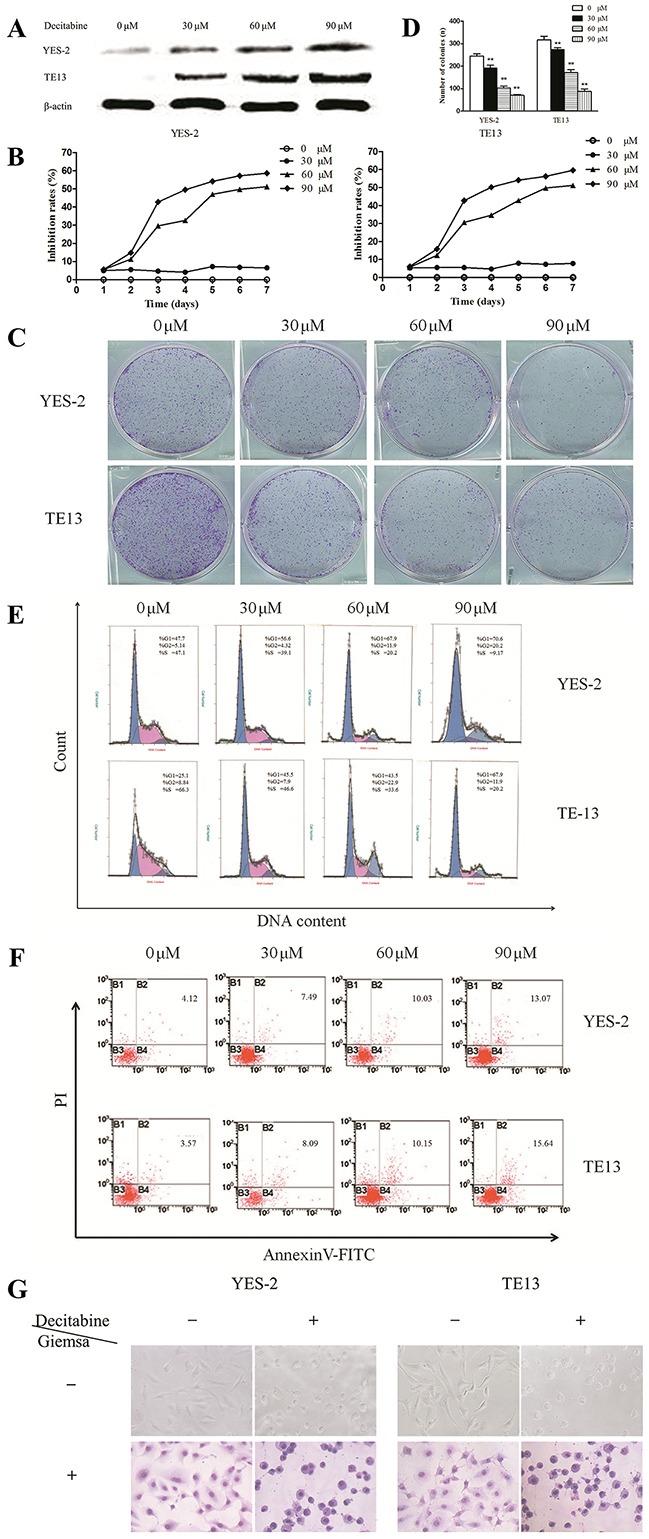
Effects of DAC-induced Bin1 de-methylation on ESCC cell proliferation, cell cycle arrest and apoptosis **A**. Protein expression of Bin1 in YES-2 and TE13 cells restored at different concentrations (0, 30, 60, and 90 μM) of DAC detected by western blot. **B**. Effect of DAC at different concentrations (0, 30, 60, and 90 μM) on the proliferation inhibition rate for 7 days investigated using MTT. The rate of proliferation was calculated (n = 3). **C**. and **D**. Significant inhibition of cell colony formation was observed upon treatment with DAC (0, 30, 60, and 90 μM) for 10 days, the number of colonies was counted and plotted on the histogram. **E**. Effect of DAC at different concentrations (0, 30, 60, and 90 μM) on the cell cycle was investigated using FCM. Representative histograms of PI stained cells. The cell cycle of the two cells arrested in the G0/G1 phase was calculated. **F**. The effect of DAC on the apoptotic rate of YES-2 and TE13 cells was detected through FCM analysis, and YES-2 and TE13 cells were treated at different concentrations (0, 30, 60, and 90 μM) of DAC. After 72 h, the cells were collected for apoptosis analysis. The percentages of annexin V- or propidium iodide-positive cells were detected using FCM. **G**. The effects of DAC on the cell morphology of YES-2 and TE13 were observed using an optical microscope with or without Giemsa staining. ** *P* < 0.01

Next, MS-PCR was used to confirm whether methylation could reduce Bin1 expression. The results showed that the Bin1 gene was completely methylated in YES-2 and TE13 cells, in which Bin1 expression was rarely detected (Figure 2B). Therefore, we used YES-2 and TE13 cells for follow-up experiments to further confirm whether the reduction of Bin1 could be reversed by DAC, a de-methylation agent that inhibited DNA methyltransferase. To this end, we treated YES-2 and TE13 cells with different concentrations of DAC. Western blot analysis and qRT-PCR showed that 30, 60, and 90 μM of DAC-restored Bin1 expression at the protein and mRNA levels in YES-2 and TE13 cells compared with those of control cells, suggesting that the restoration effect of Bin1 by DAC was dose-dependent (*P* < 0.01) (Figure 3A).

As mentioned above, the loss of Bin1 expression was associated with the hypermethylation of Bin1; thus, we detected the changes in cell proliferation, cell cycle arrest and apoptosis to confirm the biological functions of Bin1 methylation. As expected, 30, 60, and 90 μM inhibited the proliferation of YES-2 and TE13 cells compared with control cells (*P* < 0.01) (Figure 3B), and as the concentration increased, the cell growth rate decreased. Subsequently, clone formation was detected to further explore the role of Bin1 de-methylation in cell proliferation. The results indicated that the efficiency of clone formation was clearly inhibited in DAC-treated YES-2 and TE13 cells compared with control cells (*P* < 0.01) (Figure 3C and 3D). In addition, to confirm the effect of Bin1 de-methylation on the cell cycle, we determined cell cycle distributions of control *vs*. YES-2 and TE13 treated with different concentrations of DAC using FCM. Treatment with 30, 60, and 90 μM of DAC obviously induced cell cycle arrest at the G0/G1 phase and decreased cell cycle arrest at the S phase (*P* < 0.01) (Figure 3E), and the effect of DAC on cell cycle arrest was dose-dependent.

To elucidate the effect of DAC-induced Bin1 expression on cell apoptosis, Annexin V/PI staining and FCM were employed. Analysis of the proportion of apoptotic cells on the basis of FCM demonstrated that apoptosis levels in YES-2 and TE13 cells treated with 30, 60, and 90 μM of DAC, respectively, were significantly higher than those of control cells (*P* < 0.01) (Figure 3F). We also observed the changes in cell morphology in DAC-treated cells, and the results showed that compared with control cells, the DAC-treated cells were round, deeply stained nuclei, cytoplasm condensed chromatin into a lump, and the cell surface was a “budding” phenomenon, suggesting that ESCC cells were apoptotic after DAC treatment (*P* < 0.01) (Figure 3G). Taken together, these results revealed that the DAC-induced Bin1 restoration had obvious biological functions on ESCC cells.

### DAC-induced Bin1 expression prompted epithelial features and inhibited cell migration and invasion of ESCC cells

**Figure 4:**
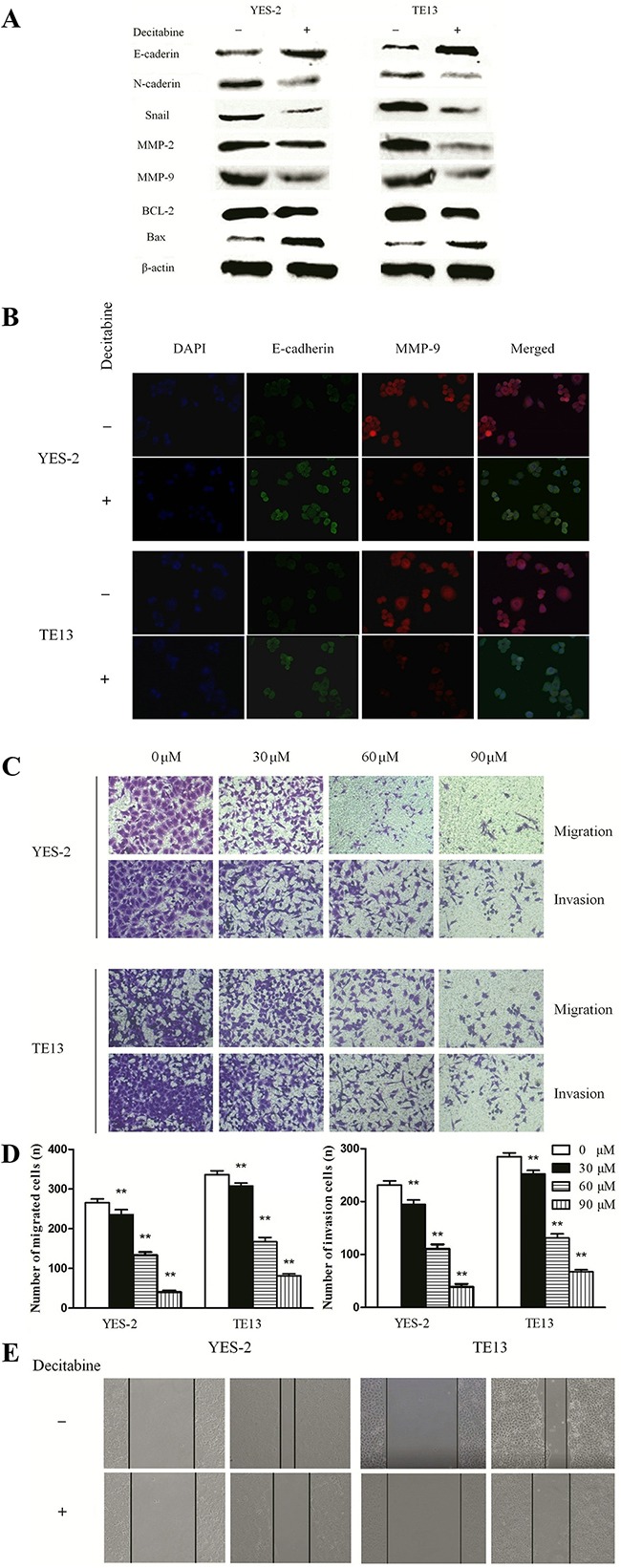
DAC-induced Bin1 de-methylation on migration, invasion and EMT of TE-13 and YES-2 cells **A**. The protein level of EMT and invasion related proteins E-cadherin, N-cadherin, Snail, MMP-2, MMP-9 and the protein level of the apoptotic-related proteins, BCL-2 and Bax were detected by western blot. β-actin served as a internal control. **B**. Immunofluorescence microscopy to identify invasion-promoting molecule MMP-9 and EMT-protective molecule E-cadherin in YES-2 and TE13 cells treated with DAC. **C**. and **D**. Effects of DAC in TE-13 and YES-2 cells with different concentrations (0, 30, 60, and 90 μM) on invasion ability exposure for 24 h were investigated using the transwell assay. The number of invading cells was calculated (n = 3). **E**. Effects of DAC at 90 μM on the migrating ability of TE13 and YES-2 cells after exposure for 48 h were investigated by wound healing assay. ** *P* < 0.01.

As described above, the data from clinical specimens indicated that Bin1 methylation was associated with the invasive and metastatic characteristics of ESCC. EMT is one of the major processes in cancer metastasis and advance by improving the migration and invasion abilities of cancer cells [14, 15]. To understand whether Bin1 de-methylation directly inhibited the EMT-induced invasion and migration of ESCC cells, we detected the surface markers and phenotypic changes of DAC-treated ESCC cells. We treated YES-2 and TE13 cells with 90μM of DAC, and the qRT-PCR results showed DAC-restored expression of Bin1 remarkably inhibited the expression of mesenchymal markers (N-cadherin and Snail) and significantly up-regulated the expression of epithelial markers (E-cadherin) in YES-2 and TE13 cells (Figure 4A). In addition, the western blot results also revealed that DAC-induced expression of Bin1 in YES-2 and TE13 cells inhibited typical EMT-like phenotypes, including the down-regulation of mesenchymal markers N-cadherin and Snail, and up-regulation of the epithelial markers E-cadherin. Immunofluorescence microscopy was employed to further determine whether Bin1 de-methylation could inhibit EMT and the migration, invasion of ESCC cells. MMP-9 and E-cadherin is primarily located in the cytoplasm and cytomembrane, with little distribution in the nucleus. After DAC treatment, the expression of E-cadherin increased, whereas MMP-9 expression significantly decreased in YES-2 and TE13 cells. These results demonstrated that, compared with control cells, DAC-treated YES-2 and TE13 cells presented a decreased level of invasion-promoted molecule MMP-9 and an increased level of EMT-protective molecule E-cadherin (*P* < 0.01) (Figure 4B).

Further, we also evaluated the effects of Bin1 de-methylation on the migration and invasion of ESCC cells using a transwell assay and a wound healing experiment. The migration assay showed that the numbers of cells migrating into transwell filters after treatment with DAC at concentrations of 0, 30, 60, and 90 μM were 206±35, 137±29, 78±18 and 39±9 of YES-2, 289±42, 145±33, 57±16 and 48±12 of TE-13, respectively (*P* < 0.01) (Figure 4C and 4D). In the wound healing assay, the cell migration rate was significantly decreased in DAC cells treated with 90 μM compared with control cells (*P* < 0.01) (Figure 4E).

Various studies have demonstrated that Bin1 might inhibit malignant activities via multiple mechanisms [9, 16, 17], thus, we detected expression alterations of invasion-related molecules MMP-2 and MMP-9 to identify potential mechanisms. The results showed that, compared with control groups, MMP-2 and MMP-9 were remarkably decreased in DAC-treated groups (*P* < 0.01) (Figure 4A). Furthermore, we evaluated the expression changes of apoptosis-associated molecules BCL-2 and Bax and observed that BCL-2 was significantly decreased and Bax was significantly increased in DAC-treated groups compared with control groups, significantly (*P* < 0.01) (Figure 4A). In conclusion, the de-methylation of Bin1 inhibited the malignant activities of ESCC cells via inactivating invasion-related molecules MMP-2 and MMP-9, apoptosis-related molecule BCL-2 and activating apoptosis-associated molecule Bax.

### DAC inhibited the malignant behaviors of ESCC cells via PTEN/AKT signaling pathways, and inhibited the Bin1 methylation induced carcinogenesis *in vivo*

**Figure 5:**
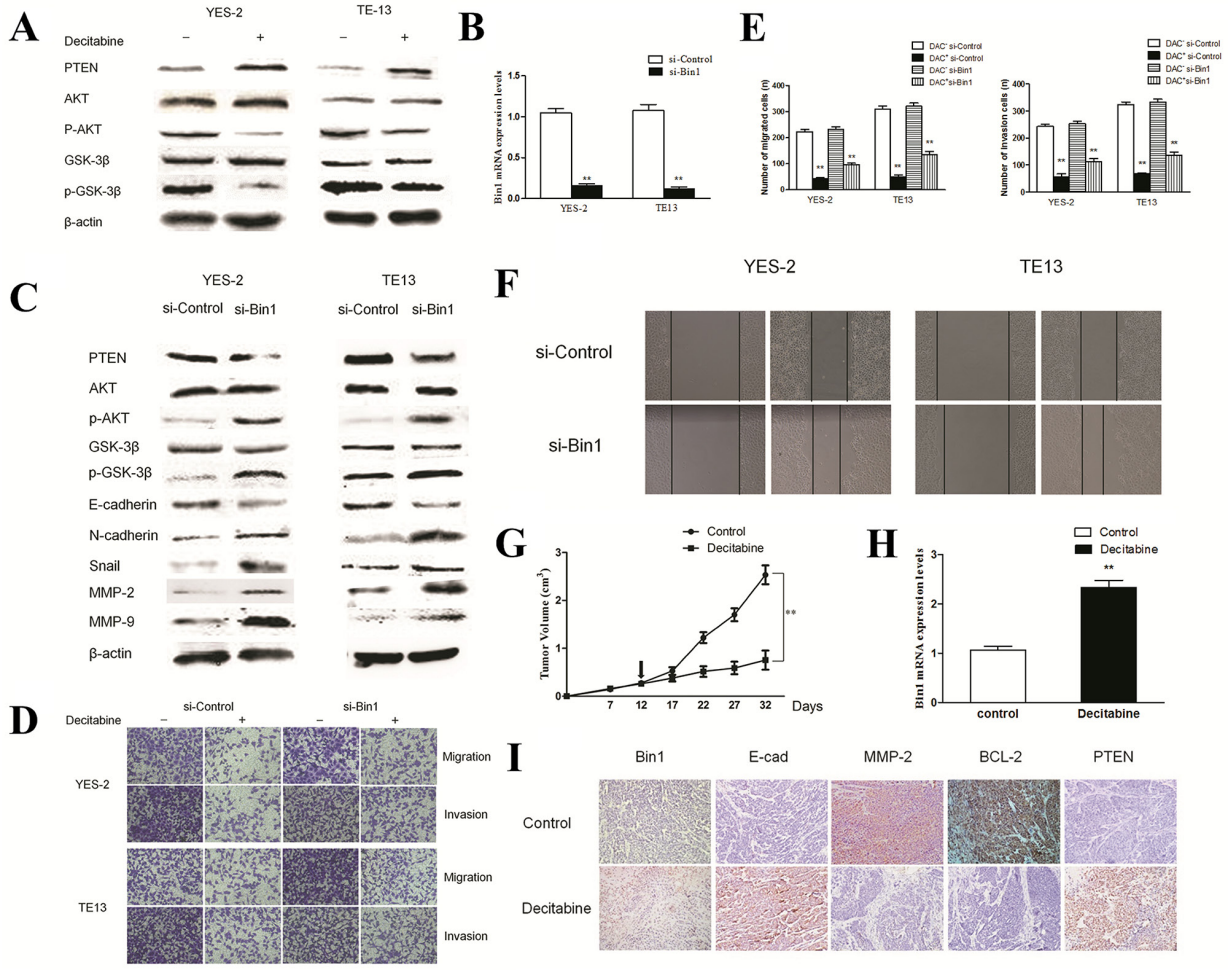
Effects of DAC on PTEN/AKT pathway in TE13 and YES-2 cells **A**. Effect of DAC on the PTEN/AKT pathway. YES-2 and TE13 cells were treated with DAC at concentrations of 90 μM. The expression of PTEN, AKT and GSK-3β proteins were detected by western blot. β-actin was used as an internal control. The figures are representative examples of three independent experiments. **B**. Effect of Bin1-siRNA on the expression of Bin1 mRNA expression in DAC-treated YES-2 and TE13 cells by qRT-PCR. **C**. Effect of Bin1-siRNA on the expression of PTEN and AKT proteins in DAC-treated YES-2 and TE13 cells. DAC-treated (90 μM) YES-2 and TE13 cells were transfected with Bin1-siRNA. The related proteins were detected by western blot. β-actin was used as an internal control. The figures are representative examples of three independent experiments. **D**. and **E**. Effect of Bin1-siRNA on the migration and invasion of DAC-treated YES-2 and TE13 cells. DAC-treated (90 μM) YES-2 and TE13 cells were transfected with Bin1-siRNA. After 48 h, the migration and invasion were observed using a transwell migration assay. The number of invading cells was calculated (n = 3). **F**. Effect of Bin1-siRNA on wound healing of DAC-treated YES-2 and TE13 cells for 48 h. DAC-treated (90 μM) YES-2 and TE13 cells were transfected with Bin1-siRNA. **G**. The tumor volume of nude mice with or without DAC. **H**. The expression level of Bin1 mRNA in nude mice with or without DAC. **I**. Representative immunohistochemistry images showing expression statuses of Bin1, E-cadherin, MMP2, BCL-2 and PTEN in tumor tissues of nude mice received different treatment (×200). ** *P* < 0.01.

Because the PTEN/AKT signaling pathways adjust malignant behaviors and is frequently initiated in cancers embracing ESCC [18], we examined the relationship between Bin1 methylation and the key molecules of this signaling pathway by using immunoblotting studies. The results showed an increase in the level of PTEN and a decrease in the levels of p-AKT and p-GSK-3β (*P* < 0.01) (Figure 5A) in YES-2 and TE13 cells compared with control cells.

As described above, treatment with DAC in ESCC cells could downregulate the major molecules of the PTEN/AKT signaling pathway. To further clarify these results, we examined whether the siRNA-mediated knockdown of Bin1 in DAC-treated YES-2 and TE13 cells could reactive PTEN/AKT signaling pathways. Silencing Bin1 with Bin1-siRNA in DAC-treated YES-2 and TE13 cells increased the expression of p-AKT and p-GSK-3β and decreased the expression of PTEN compared with control cells (*P* < 0.01) (Figure 5B and 5C). Thus, the PTEN/AKT signaling pathway could be affected to suppress the malignant activities of ESCC cells via DAC-induced Bin1 expression.

We detected the EMT, migration and invasion of ESCC cells after reactivation of the PTEN/AKT signaling pathway. Knockdown of Bin1 in DAC-treated YES-2 and TE13 cells increased the expression of mesenchymal markers (N-cadherin and Snail) and inhibited the expression of epithelial markers (E-cadherin) at the protein level (Figure 5C). Moreover, transwell migration and wound healing assays also indicated that Bin1 knockdown using siRNA significantly promoted the migration and invasion of DAC-treated YES-2 and TE13 cells (Figure 5D, 5E and 5F). Taken above, inhibition of Bin1 expression and reactivation of the PTEN/AKT signaling pathway in ESCC cells promoted EMT-like molecular changes and facilitated the migration and invasion of ESCC cells.

To identify the effect of DAC on Bin1 methylation *in vivo*, we injected YES-2 cells harboring fully methylated Bin1 into null mice and detected the tumor volume after 12 days. The results showed that the average tumor volume of DAC-treated mice was significantly smaller than that of the control group (*P* < 0.01) (Figure 5G). In addition, the Bin1 expression in ESCC tissues of null mice was significantly increased compared with the control group (*P* < 0.01) (Figure 5H). All tumors formed in DAC-treated mice presented strong Bin1 staining (*P* < 0.05) (Figure 5I), suggesting that DAC could reverse the carcinogenesis via the de-methylation of Bin1.

Furthermore, to verify the mechanisms of tumor inhibition by Bin1 *in vivo*, we detected the expression alterations of MMP-2, E-cadherin and BCL-2 in tumor-bearing mice using IHC. The results demonstrated that, compared with control group, the DAC-treated mice presented decreased MMP-2 and BCL-2 and increased E-cadherin (*P* < 0.05). Significantly, we also evaluated the expression changes of the major molecules of the PTEN/AKT signaling pathway, the results showed that DAC-treated mice presented increased PTEN expression (*P* < 0.05) (Figure 5I). These results indicated that DAC treatment could restore the tumor suppressing functions of Bin1 to suppress malignancy-associated molecules and the PTEN/AKT signaling pathway *in vivo*.

## DISCUSSION

ESCC is one of the most common malignant cancers with disenchanting prognosis. Previous studies have demonstrated that the silencing of tumor-protective signature genes is one of the major causes of ESCC carcinogenesis and progression [4]. Bin1, which can function as a tumor suppressor, shows low expression in many malignant carcinomas, including ESCC, but its associated regulation mechanisms, particularly the epigenetic mechanisms, remain elusive. Therefore, identifying the involved mechanisms is essential for improving clinical treatment.

The silencing of tumor suppressor genes by DNA methylation is one of the mechanisms for most aggressive neoplasms and the poor overall prognosis in cancer patients [19]. Numerous studies reported that DNA methylation also contributed to the progression of ESCC through numerous ways, such as inducing EMT [20–22]. In the present study, we found that the Bin1 gene was hypermethylated in ESCC tissues, accounting for the reduction of Bin1 protein. Significantly, the methylation status of Bin1 was remarkably associated with poor differentiation grade, high TNM stage, deep tumor invasion, positive lymph node metastasis, and poor PFS and OS, indicating that the hypermethylation of Bin1 could be regarded as an independent predictor of the poor prognosis of ESCC patients.

Bin1 is a conserved member of the BAR (Bin-Amphiphysin-Rvs) family of adapter proteins participating in diverse cellular processes including endocytosis, programmed cell death, DNA repair, stress responses, and transcriptional control [17, 23]. Several studies have indicated Bin1 reduction is a key factor in driving the progression of certain types of tumors, such as childhood neuroblastoma (NB) and breast cancer [24–26]. In this study, we observed a hypermethylation status of Bin1, which contributed to the attenuated Bin1 expression in ESCC cells, and the de-methylation agent Decitabine could restore the expression of Bin1 by reversing this hypermethylation. Bin1 is a Myc-interacting adaptor protein with tumor suppressor characteristics, including the suppression of Myc-mediated cell malignant transformation and proliferation [24]. In addition, the loss of Bin1 expression is associated with various tumor biological characteristics, such as lymph node metastasis and cell apoptosis resistance [27, 28]. Zhong et al. showed that Bin1 might function as a metastasis inhibitor and chemosensitizer in NB by neutralizing resistance to anoikis [29]. The present study indicated that restoring Bin1 expression by Decitabine treatment in ESCC cells could inhibit cell proliferation, arrest the cell cycle and promote cell apoptosis. Therefore, the tumor-side injection of DAC could restore Bin1 expression and inhibit the carcinogenesis of ESCC in null mice.

Recently, EMT has been shown to play a significant role in facilitating metastasis and in epithelium-derived tumors. Energizing EMT is necessary to promote cell-cell junction loss and separate tumor cells for single-cell migration and invasion. For example, Derksen et al. showed that mice carrying a genetic loss of E-cadherin gene on a mammary-specific p53-null background developed invasive lobular carcinomas, a subtype of breast cancer that presents with individual migrating tumor cells [30–33]. The EMT program is included in the ECM (extracellular matrix) process via up-regulation multiple matrix degradation enzymes by the EMT core regulators. Snail expression in MCF-7 breast cancer cells increased MMP-9 expression and promoted the breakdown of basement membrane [34]. Recent evidence suggests that EMT transcription factors can also cause the formation of specialized subcellular structures, called invadopodia, to invade local ECMs [34]. The present study indicated that DAC-restored Bin1 expression could inhibit the cell migration, invasion and EMT of ESCC cellss.

The PTEN/AKT cascade is a central pathway regulating diverse processes, such as metabolism, cell proliferation, migration and apoptosis [35, 36]. To explore the potential tumor suppressing mechanisms of Bin1 in ESCC, we detected the expression of the PTEN/AKT signaling pathway and found that decreased p-AKT and increased PTEN were associated with cell migration, invasion and EMT both *in vitro* and *in vivo*. Moreover, transfected *Bin1*-siRNA in DAC-treated YES-2 and TE13 cells could reactivate the PTEN/AKT pathway. These results demonstrated that Bin1 could inhibit ESCC cell migration, invasion and EMT by inactivating the PTEN/AKT signaling pathway. Furthermore, MMP-2 and MMP-9 can break down collagen, which is an important component of the basement membrane, and are significantly related to cancer invasion and metastasis [37]. These studies indicated that DAC-restored Bin1 expression could inhibit cell migration and invasion by suppressing MMP-2 and MMP-9 expression.

In conclusion, despite the existing studies on Bin1 methylation status in some cancers, this study is the first to identify its methylation status and biological functions in ESCC. These results demonstrated that the Bin1 methylation, primarily accounting for Bin1 attenuated expression, was closely related to the poorer clinicopathological characteristics and the worse survival of ESCC patients. Bin1 methylation could promote the malignant behaviors including EMT of ESCC cells *in vitro* and *in vivo*. Therefore, de-methylation treatment with Decitabine could neutralize these malignant activities including EMT by restoring Bin1 expression. Moreover, we also observed that Bin1 could inhibit EMT and invasion via inactivating PTEN/AKT signaling pathway and suppressing MMP-2 and MMP-9. The present study revealed the potential role of Bin1 methylation in ESCC carcinogenesis, suggesting that the DAC-based restoration of Bin1 could be a potential therapeutic strategy for improving the prognosis of ESCC patients.

## MATERIALS AND METHODS

### Patients and specimens

The specimens of ESCC tissues were collected from 116 patients who underwent esophageal cancer surgery at the Fourth Hospital of Hebei Medical University (Shijiazhuang, China) between May 2011 and May 2012. This research was approved through the ethic committee of the Hebei Medical University and informed consent was obtained from all patients. The median patient age at the time of surgery was 58 years (range: 33-71 years). None of the ESCC patients received radiotherapy, chemotherapy and immunotherapy before operation. The clinical stage and histological tumor type were determined according to the UICC Classification of 2009 (seventh edition) and the WHO classification of 2005. Patient's clinical information was collected and stored in a database, which was updated every 3 months by telephone follow-up. Complete follow-up was updated until death or May 2016.

### Materials

RPMI-1640 and fetal bovine serum (FBS) were obtained from Gibco-BRL (Life Technologies, Paisley, Scotland). Antibodies to total PTEN, AKT, p-AKT, BCL-2, Bax, E-cadherin, N-cadherin, Snail, matrix metalloproteinase (MMP)-2, and MMP-9 were all purchased from Cell Signaling Technology, Inc. (Danvers, CA, USA); Antibodies to Bin1 and β-actin were purchased from Abcam, Inc. (Cambridge, MA, USA). Go Taq® qPCR Master Mix was purchased from Promega (Madison, WI, USA). RevertAid™ First Strand cDNA Synthesis Kits was purchased from MBI Fermentas (Hanover, MD, USA). Annexin V fluorescein isothiocyanate (FITC) and PI double stain was purchased from BD Pharmingen (San Diego, CA, USA).

### Cell lines

The esophageal cancer cell lines TE-13, TE-1, EC109, KYSE30, YES-2 and human normal esophageal epithelial cell (HEEC) were obtained from the Research Center of the Fourth Hospital of Hebei Medical University (Shijiazhuang, China). The cells were seeded at a low density and incubated for 24 h prior to treatment with the DNA methylation transferase inhibitor Decitabine (DAC). The cells were treated with DAC (Sigma, St Louis, MO, USA) for 72 h, and DAC-containing media were changed every 24 h.

### Immunohistochemical assay

IHC analysis was performed using the streptavidin-peroxidase (SP) method. The rabbit polyclonal antibody against human Bin1, BCL-2, and PTEN at a dilution of 1:100 and MMP-2 and E-cadherin at a dilution of 1:50 were used for detection and incubated at 4°C overnight. The sections were further incubated with biotinylated secondary antibody and ABC reagent. The staining was visualized and classified based on the percentage of positive cells and the intensity of staining according to a 0-4 semi-quantitative system. The total scores were determined after multiplying the percentage and intensity scores and graded as low for a score of 0-4 and high for a score of 5-12.

### Cell viability assay

The effect of DAC on ESCC cell viability was determined using the MTT reduction (3-(4,5-dimethylthiazol-2-yl)-2,5-diphenyltetrazolium bromide) assay according to the manufacturer's instructions (Promega, Madison, WI). MTT can be reduced to formazan by cytoplasmic and mitochondrial reductases present in viable cells. A total of 1×10^4^ TE13 and YES-2 cells per well treated with DAC (0, 30, 60, and 90 μM) were additionally treated with 3 mg/mL MTT (Sigma-Aldrich, St. Louis, USA) and measured using a microplate reader to assess the inhibition rate of DAC on cell viability.

### Flow cytometry assay (FCM)

The effect of DAC on apoptosis was assessed through the FCM analysis of cells incubated with Annexin V-FITC and PI double staining according to the manufacturer's instructions. Cell cycle analysis was performed using PI staining. Single-cell suspensions of TE13 and YES-2 cells (1×10^6^ cells per sample) were stained with PI for 15 min and analyzed using fluorescence activated cell sorting (FACS) flow cytometer (FACS Caliber™, Becton-Dickerson, USA). The data were analyzed using CellQuest Pro software.

### Cell morphology

Wright-Giemsa staining was used to evaluate the effect of DAC on cell cytomorphological alterations. A total of 5×10^4^ TE13 and YES-2 cells were cultured in 6-well plates, after treatment with 90 μM of DAC for 72 h, and the cells were fixed and stained with Wright-Giemsa for 30 min.

### Methylation specific PCR assay

A methylation-specific polymerase chain reaction PCR (MS-PCR) assay was performed in a total volume of 20 μl using 1.5 units Platinum Taq-polymerase (Invitrogen) per reaction. Oligonucleotide sequences used for the MSP were: *Bin1* U forward: 5′-GGAAGATTAAGATGTTAGGTTGGTG-3′; *Bin1* U reverse: 5′-ACCAAACCTCAAACCACAATCAACA-3′; *Bin1* M forward: 5′-GGAAGATTAAGACGTTAGGTCGGCG-3′; *Bin1* M reverse: 5′-ACCGAACCTCAAACCGCGATCGACG-3′.

### RNA preparation and quantitative reverse transcription polymerase chain reaction (qRT-PCR) analysis

The expression of *Bin1* mRNA in tumor tissues, ESCC cell lines and nude mouse model were detected by qRT-PCR analysis. Total RNA was extracted using TRIzol reagent (Invitrogen, Shanghai, China) according to the manufacturer's instructions. First-strand cDNAs were generated, and the cDNAs were amplified by qRT-PCR using Go Taq® qPCR Master Mix (Promega, Madison, WI, USA) and specific primers for *Bin1* and *β-actin* (Shanghai Generay Biotech), with the latter used as an internal control. *Bin1* Forward : 5′-CAAGTCCCCATCTCAGCCAG-3′, Reverse : 5′-GGATCACCAGCACCACATCA-3′, *β-actin* Forward : 5′-GTCACCTTCACCGTTCCAGTTTT-3′, Reverse : 5′CTTAGTTGCGTTACACCCTTTCTT-3′.

### Colony formation assay

To investigate the effects of DAC on the colony formation of esophageal carcinoma cells, a colony-forming assay was performed. Briefly, 500 TE13 and YES-2 cells were seeded for 10 days, and subsequently, the cell colonies were stained with crystal violet (2%) for 10 min and counted.

### Tumor cell migration and invasion assays

Tumor cell migration assay was performed in a 24-well transwell chamber (Collaborative Biomedical, Becton Dickinson Labware, Bedford, MA), containing an 8-μm pore size polycarbonate membrane filter precoated with 100 μg of Matrigel for invasion assay (Becton-Dickinson, Bedford, USA). A total of 2×10^5^ cells treated with 0, 30, 60, and 90 μM DAC were seeded in the upper chambers and incubated for 24 h. The cells on the lower side of the filters were defined as migration cells and counted.

The migration of tumor cells was also assessed using a wound healing assay. TE-13 and YES-2 cells were seeded at 2×10^4^/well. After scraping the cell monolayer with a sterile micropipette tip for 48 h, each scratch was examined and captured at the same location, and the healed area was measured.

### Immunofluorescence

YES-2 and TE13 cells were harvested and incubated with rabbit to human MMP-9 and mouse to human E-cadherin mAb at 4°C overnight. The cells were subsequently stained with FITC-conjugated goat anti-mouse and PE-conjugated goat anti-rabbit antibodies, followed by DAPI staining of the nucleus. The fluorescence was observed and analyzed using a fluorescence microscope at high magnification (×400).

### Western blot analysis

TE-13 and YES-2 cells were lysed and subjected to sodium dodecyl sulfate polyacrylamide gel electrophoresis (SDS-PAGE), followed by incubation with different dilutions of the primary antibodies, including antibodies to MMP-2, MMP-9, Bin1, PTEN, AKT, p-AKT, BCL-2, Bax and β-actin. The levels of protein in each sample were normalized relative to those of β-actin.

### Silencing Bin1 expression in DAC-treated YES-2 and TE13 cells

To further investigate the mechanisms of tumor inhibition by Bin1, the *Bin1* gene was silenced with siRNA. A total of 400 pmol of siRNA was transfected into 4×10^5^ DAC-treated YES-2 and TE13 cells using Lipofectamine RNAi MAX reagent (Invitrogen, NY, USA) according to the manufacturer's protocol. Subsequently, the expression changes in PTEN/AKT signaling pathways were detected.

### *In vivo* tumor growth assay

Balb-c/null mice were used in this vivo tumor growth assay. YES-2 cells (1×10^6^ cells/mouse) in 0.1 ml were subcutaneously injected into Balb-c/null mice. The mice were randomly divided into two groups (6 mice/group): GI: 95% Ethanol group; GII: DAC (1 mg/kg) group, treated once per two days. All mice received treatment on day 12 and were sacrificed by cervical dislocation on day 32. IHC staining was performed to detect the expression of Bin1, E-cadherin, MMP-2, BCL-2 and PTEN in tumor tissues.

### Statistical analysis

Data are reported as the means ± SD. One-way analysis of variance (ANOVA) was performed to determine the significance between groups. Tukey's method was used for multiple comparisons. A *P*-value of less than 0.05 and 0.01 was considered statistically significant. The data were obtained from at least three independent experiments with a similar pattern. All data analyses were performed using SPSS 21.0 software.
